# Automatic Realistic Real Time Stimulation/Recording in Weakly Electric Fish: Long Time Behavior Characterization in Freely Swimming Fish and Stimuli Discrimination

**DOI:** 10.1371/journal.pone.0084885

**Published:** 2014-01-06

**Authors:** Caroline G. Forlim, Reynaldo D. Pinto

**Affiliations:** 1 Departamento de Física Geral, Universidade de São Paulo, São Paulo, SP, Brazil; 2 Laboratório de Neurodinâmica/Neurobiofísica, Universidade de São Paulo, São Carlos, SP, Brazil; Centre national de la recherche scientifique, France

## Abstract

Weakly electric fish are unique model systems in neuroethology, that allow experimentalists to non-invasively, access, central nervous system generated spatio-temporal electric patterns of pulses with roles in at least 2 complex and incompletely understood abilities: electrocommunication and electrolocation. Pulse-type electric fish alter their inter pulse intervals (IPIs) according to different behavioral contexts as aggression, hiding and mating. Nevertheless, only a few behavioral studies comparing the influence of different stimuli IPIs in the fish electric response have been conducted. We developed an apparatus that allows real time automatic realistic stimulation and simultaneous recording of electric pulses in freely moving *Gymnotus carapo* for several days. We detected and recorded pulse timestamps independently of the fish’s position for days. A stimulus fish was mimicked by a dipole electrode that reproduced the voltage time series of real conspecific according to previously recorded timestamp sequences. We characterized fish behavior and the eletrocommunication in 2 conditions: stimulated by IPIs pre-recorded from other fish and random IPI ones. All stimuli pulses had the exact *Gymontus carapo* waveform. All fish presented a surprisingly long transient exploratory behavior (more than 8 h) when exposed to a new environment in the absence of electrical stimuli. Further, we also show that fish are able to discriminate between real and random stimuli distributions by changing several characteristics of their IPI distribution.

## Introduction

Weakly electric fish offer a unique opportunity to non-invasively study and interact with living intact nervous systems. Their electric organ discharges (EODs) are complex spatio-temporal signals involved in processing a plethora of environmental sensory information [1]–[4].

Electric signal production in pulse-type weakly electric fish has 2 basic components: the waveform and the timing of the electric pulses [1]. The pulse waveforms are species-specific and the inter EOD intervals or inter pulse intervals (IPIs) can vary from tens to hundreds of milliseconds [5]. Each EOD generates electric fields that propagate into the water, interact with the surroundings and reach a myriad of electroreceptors disposed to detect electric field changes that cause small alterations in self or conspecific transcutaneous currents [2], [5].

Fish are able to localize and measure the distance of nearby objects with different impedance in the surrounding water by analyzing the alterations in the self-generated electric field; this process is called active electrolocation [4], [6]–[10].

They can also perform electrocommunication; in this process, 1 fish’s EODs are detected by another fish’s receptors. During this process, each fish emits and receives self and conspecific EODs [11]–[13]. The neuroethology of electrolocation and electrocommunication remain interesting and active fields of research [8], [14]–[16]. Electrocommunication is considered to be an important factor in brain evolution and a diversification trigger in electric fish [17].

The complex spatio-temporal pattern generated by the EODs makes experimentation difficult in freely moving fish [18]. For this reason, many previous experiments to characterize the electrosensory system were conducted by shortly moving fish to shallow tanks, restraining their movements, despite the sensitivity of the fish IPI patterns to stimuli, environment and manipulation. Studies of electrical interactions between fish were possible in species that presented an accentuated sexual dimorphism in EOD shape during breeding season [19], [20], or during resting behavior [18], [21], or in situations where specimens of very different sizes were used [18], [21], [22]. These parameters allowed easier separation of fish’s EODs from the stimuli ones; otherwise, reliable separation of the EODs depends on the fish’s relative orientation and proximity to the electrode, making automatic real-time detections very difficult [22] due to the changes in the measured amplitude and waveform [18], [23]. Recent advances have been made in measuring automatically freely moving non-stimulated fish [24], [25], but no general solution has been yet presented to automatically detect, stimulate and separate each fish’s EODs from conspecific’s ones.

In many pulse-type species such as *Gymnotus carapo*, the EOD of each individual is a stereotyped pulse shape with no sexual dimorphism [9]. In such species, fish can also vary their IPIs depending on the environmental context and such variations have been associated to behavioral contexts [26], [27], as well as species recognition [28]. Nevertheless, there are only a few comparative studies linking stimuli IPI distributions and patterns to fish’s electrical response [26], [27], [29], [30]. For example, when a new stimulus (light, sound, electrical field or vibration) is presented a transient EOD rate acceleration, called the novelty response (NR) [31]–[34] is triggered. A jamming avoidance response (JAR) mechanism has also been reported in which fish adjust their EOD timing to avoid discharging at the same time [35], [36].

Entropy is a powerful tool to identify and analyze relevant changes in the complex spatio-temporal pattern discharged by the OE and relate such changes with fish behavior. It is a measure of uncertainty or unpredictability and characterizes how much information is available. Entropy has been widely used in neural systems [37], [38], [39], [40] and can be applied to adaptive systems with complex non-linear dynamics. It is defined as [41]:
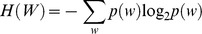
where *w* is the outcome, *p* is the probability and *H* is entropy

When an outcome is highly predictable, the entropy is low. That is, if fish discharge the same EOD patterns, the entropy for such a signal would be zero.

One can compute the entropy of any time series, however, to relate fish IPI entropy with behavior we must ensure that the process of recording the EODs produce the minimum disturbance possible to free-swimming animals.

We show an apparatus that allows non-invasive realistic and controlled electrical stimulation with simultaneous recording of EOD timestamps for several days of freely moving pulse-type fish *Gymnotus carapo*. The apparatus is based on a simple and inexpensive circuitry than can be easily adjusted for different species of electric fish [42], [43], [44], to fieldwork, and to connect and stimulate 2 fish in real time.

We concentrated our experiments in 2 different situations: (1) non stimulated fish and (2) stimuli from an artificial fish that mimics the pulse shape of a conspecific. We could evaluate how long the stress caused by exposing the fish to a new environment distorts the patterns of electrical activity, the relation, between the locomotor and electrical activity the differences in the electrical activity when the fish are exposed to real (conspecific) or random distributions of stimuli IPIs.

## Materials and Methods

### Ethics Statement

All experimental protocols and procedures were in accordance with the ethical principles of the Society for Neuroscience and were approved by the Committee on Ethics in Animal Experimentation of the Federal University of São Carlos – UFSCar (Protocol number 007/2009).

### Animals

Adult specimens, 15–25****cm long, of unsexed *Gymnotus carapo* were acquired from local fishermen in the state of São Paulo, Brazil. The fish were maintained in individual 31.5 L (30×35×30 cm) unplanted tanks, exposed to natural illumination at room temperature and provided with lengths of polyvinyl chloride (PVC) pipe as hiding places. Water conductivity was 100±5 µS/cm; similar value found in the streams or lagoons where these fish are usually captured. Fish were fed a variety of living food twice a week including: small fish, worms, and *Artemia salina* (brine shrimp). In total, 20 fish were used in the reported experiments, and recordings of some fish were obtained several times in different protocols.

### Measurement Aquarium

The experimental setup, developed in our laboratory [45]–[47] consisted of glass aquarium (40×40×45 cm) filled with 64 L of water (40×40×40 cm; and enclosed in a double Faraday cage (1 mm aluminum sheets) to shield from electrical noise and in two 3 cm thick layers of insulating foam confining a 4 cm air layer to attenuate sound waves. Other potential low amplitude, low frequency mechanical vibrations were attenuated by hanging the apparatus from steel cables attached to the ceiling.

Fish were not fed in the measurement aquarium, and the room temperature was fixed at 23°C to ensure that the water temperature and conductivity did not change during the experiments.

**Figure 1:**
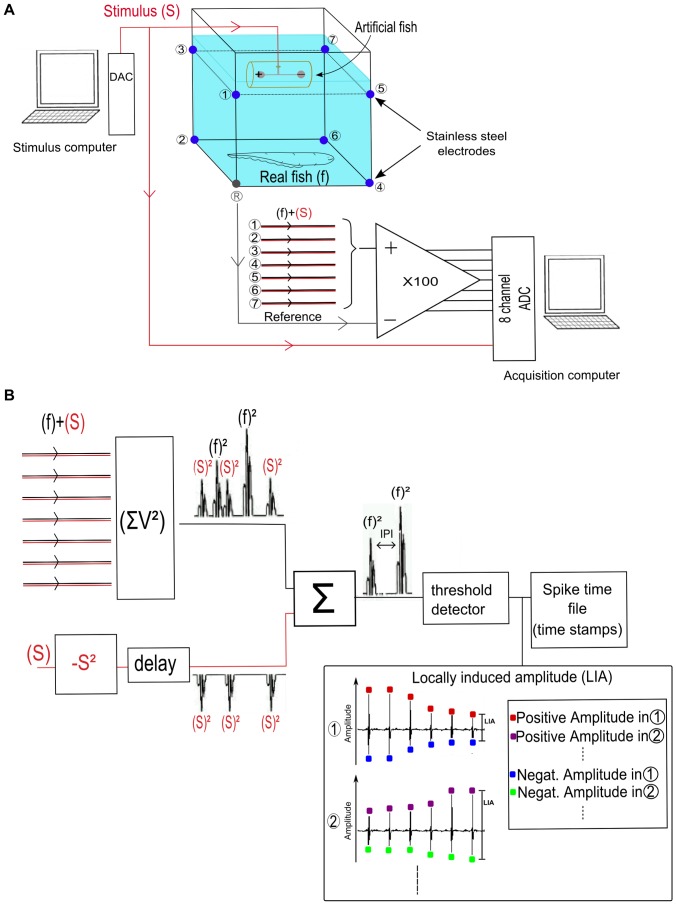
Acquisition and stimulation setup. **A – hardware:** EODs from real (f) and artificial stimulus (S) fish are measured using a 3D array of 8 electrodes [(1)-(7), and (R)]. Signals are differentially amplified (x 100, (R) is the common reference) and digitized at 50 kHz by an ADC board. The artificial fish is a dipole electrode inside a 15 cm PVC pipe driven by a DAC output from a computer running real-time home made stimulation software that mimics a real fish pulse shape with IPIs defined by the experimenter. **B – acquisition software:** the square of the digitized artificial signal are delayed of 1–3 samples due to the DAC-ADC circuitry and subtracted from the squared and summed electrode signals, because this signal contains both stimulus (S) and real fish (f) and after subtraction, only the real fish pulses will remain. Real fish pulses are then detected with a simple threshold level and the timestamps at each electrode are stored for posterior analyzes. The positive and negative amplitude values at each timestamp are detected in all electrodes. The electrode corresponding to the maximum positive amplitude indicates the position of the fish’s head and the electrode with the most negative amplitude indicates the position of the fish’s tail. If the fish’s head(tail) is in the middle of the tank, the positive (negative) amplitudes will be more or less the same in all electrodes and not as high as if the fish were near the corners of the tank or the reference electrode. The locally induced amplitude (LIA) at each electrode is defined as the difference between the maximum and minimum values of voltage induced during the EOD.

Spatio-temporal EOD was measured using a three-dimensional (3D) array of 8 electrodes, each consisting of a 0.2 mm diameter stainless steel wire tightly inserted through silicone glue, between the glass aquarium walls, dipping 1–2 mm into the water. The electrodes were placed in a cubic arrangement (∼40 cm on a side, Fig. 1A). Signals from 7 electrodes were differentially amplified (gain = 100x, home-made) using the electrode at the base of the aquarium as a common reference. The time series of all differential amplifiers (containing both EOD and artificial stimulus signals) were digitized at 50 kHz by an analog-to-digital converter (ADC) board (PCI MIO 16-E1, National Instruments, Texas) in an acquisition PC-compatible computer.

### Artificial Stimulus Generation

**Figure 2:**
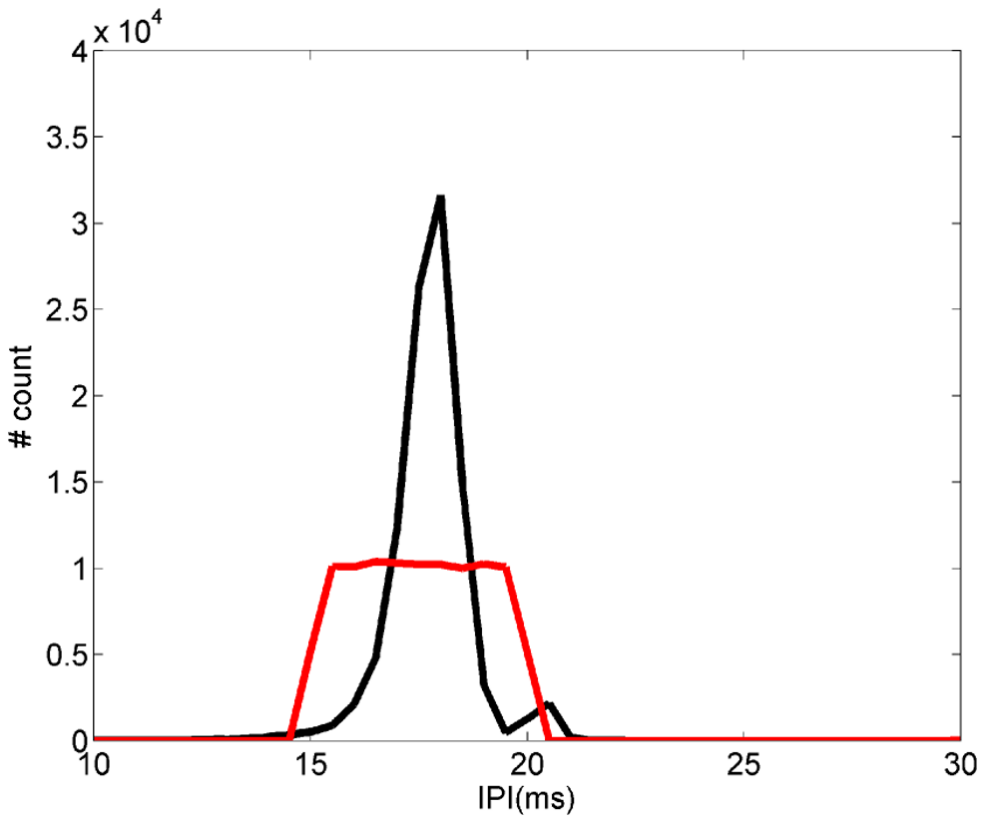
Stimuli histograms. Real IPI stimulus histogram (in black). The IPIs were recorded (30 min) from a non-stimulated fish immediately after being introduced in the tank. Random IPI stimulus histogram (in red). IPIs from 15 ms to 20 ms were generated with the same probability (flat distribution) by computer.

A dipole electrode placed inside a 15 cm PVC pipe was used to generate artificial stimuli mimicking the geometry of a medium size fish. Although *Gymnotus carapo* EODs are more complex than the signal emitted by a dipole, for this particular study it was a good approximation. Artificial fish stimuli were generated by a digital to analog converter (DAC) board (Digidata 1200B, Axon Instruments, Union City, CA), controlled by a real-time software developed by our group [47], [48] to mimic a real *Gymnotus carapo* EOD waveform, previously stored in a personal computer (PC; Fig. 1A), and rescaled to a fixed peak amplitude of 5****V. Two stimuli timestamp sequences (30 min long) could be chosen by the experimenter: one pre-recorded from an unstimulated solitary fish just after being introduced in the tank, and one with randomly generated IPIs (flat distribution) in the same range as the IPI of the real fish (15–20 ms). A flat IPI distribution means that after a given IPI, the next IPI will be between 15 ms and 20 ms, with all values in this interval chosen with the same probability, avoiding possible causalities (Fig. 2).

### Acquisition Software

*Gymnotus carapo* generates a multi-phasic EOD pulse [50]. A simple way to detect the timing of an EOD is to determine the occurrence of the major pulse component.

However, depending on the fish orientation, the major component can be either negative or positive for a given electrode, thus making detection difficult.

To overcome this problem, our detection software squared and summed the digitized time series registered by all 7 electrodes, compressing EOD information in a single time series. By comparing the compressed time series with a threshold level we were able to reliably detect the EODs (Fig. 1B), with a resolution of 20 µs. For each EOD detected, the program recorded the event timestamp and the locally induced amplitude (LIA) at each electrode, (defined here as the difference between the maximum and minimum values of voltage induced during the EOD [Fig. 1B]).

The LIA is inversely proportional to the fish-electrode distance [18], [22]; therefore, if the fish stood still for a period, the LIA would have been practically the same from pulse to pulse, providing a low standard deviation value. Conversely, the LIA amplitude would have changed from pulse to pulse in at least a few electrodes for active fish, producing higher standard deviation values for those electrodes. By summing the standard deviations over all electrodes, we estimated if the fish were swimming or standing, disregarding their position and orientation.

In experiments with artificial stimulation, the ADC input signals from the electrodes contained both real fish and artificial pulses; therefore, we subtracted the square of the artificial signal from the summed signal to avoid recording artificial pulses as real fish pulses (Fig. 1B). The squared value of stimulus+fish pulse can show a small delay compared to the artificial stimuli of 1–3 samples due to the DAC-ADC circuitry. Elimination of the artificial stimulus from the stimulus+fish pulse will be maximal when there is no delay between these 2 signals.

The detection software was written in DASYlab graphic language (Dasytech, Germany).

### Data Analyzes

Information Theory provides invaluable tools to study neural coding [41]. Here we inferred the timestamp sequences variability by calculating the entropy H(T) as a function of time, using a direct method [51] adapted to our data.

**Figure 3:**
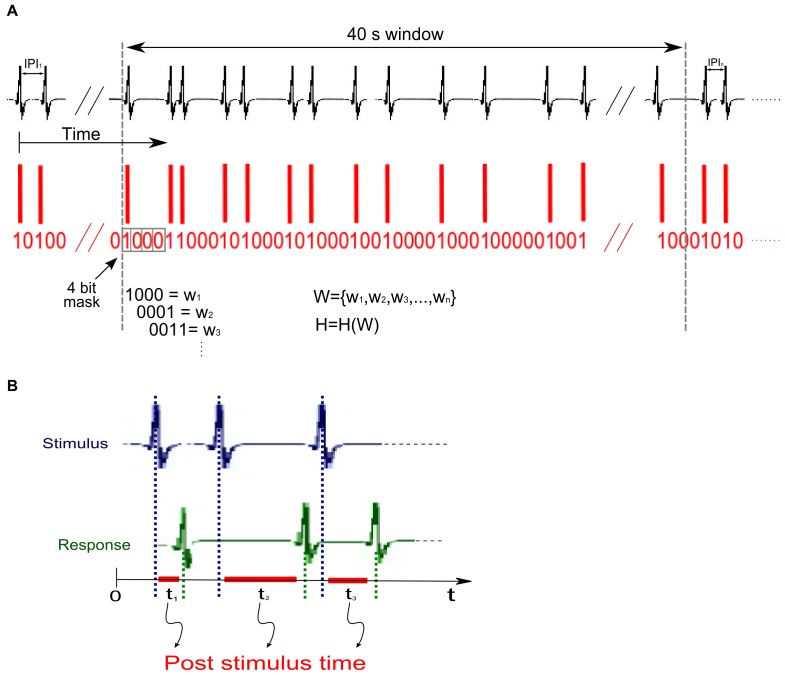
Data analyzes: binarization, entropy and post stimulus calculations. **A –** Binarization and set of patterns for entropy calculations. The time axis is discretized into small bins. The presence of an EOD in each bin is represented by 1, whereas an absence is represented by 0. Timestamp sequences are analyzed in bit strings obtained in 40*W = *{*w_1_*, *w_2_*, *w_3_*,…, *w_N_*}. From the set of probabilities of each word *w_i_* we computed the entropy *H*(W). **B –** Post stimulus time (PST) is defined as the time intervals (in red) *ti* between stimulus pulses (in blue) and the fish response ones (in green).

We analyzed the timestamp sequences in T = 40 s windows, but the same qualitative results were also found using 20 and 60 s windows. The first window spanned from 0 to 40 s, the second from 20 to 60 s, and so on, maintaining a 20 s time distance between the start of successive windows (Fig. 3). For each window, we calculated the probability of observing different EOD patterns and obtained the entropy from this probability distribution. To compute the entropy H(T), the time axis was discretized into *b* sec wide bins. The presence of an EOD in each bin was represented by 1, and the absence was represented by 0. Thus, we encoded a sequence of EODs as a binary string (Fig. 3A).

The entropy H depends on the value for b: H = Hb. If b was too small, most of the bits of our strings would be zero, and very few patterns would have been found. However, if b was too big, most bits would be set to 1, again leading to very few patterns. In both cases, the low entropy values would have not satisfactorily represented the variability of the data. Hence, an intermediate optimal value of b was required to correctly express the variability of the data. In agreement with the principle of maximization of entropy [51], choosing b such that Hb was a maximum would have produced the least structured set of patterns consistent with the data. Intuitively, suitable values of b would provide strings with similar amounts of zeros and ones. In our case, the fish average IPI was about 20–40 ms, hence b should have been of the order of 10–20 ms. To determine the “best” value of b, we computed entropy for b between 3 and 40 ms, in 1-ms steps.

Next, we decided on the length *L* of patterns, for which the probability was computed. In our analyzes each pattern was represented by a *word* composed by *L* = 4 bits. For a given *b* we started at the beginning of the corresponding string and using a 4-bit mask we copied the first 4 bits to the word *w_1_*. Then, we moved the mask 1 bit to the right and copied the bits to the word *w_2_* (Fig. 3A). This process was repeated until the mask reached the end of the window. *W = *{*w_1_*, *w_2_*, *w_3_*,…, *w_N_*} is the set of all words (patterns) from which we computed the probabilities *P*(*w*) of finding each 1 of the 2^4^ = 16 different words.

To ensure a good statistical estimation of *P*(*w*) one should use stationary data and the largest window length possible. However, the entire timestamp sequences are not stationary (some of them are several days long), thus we explore this feature by analyzing changes in the entropy along the sequence and relate them to the fish behavior. The parameter of T = 40 s was long enough to provide an average number of words N∼(40 s/15 ms)∼2500 words, giving a good statistical value of *P*(*w*) for all possible 16 words and still capturing the fish behavior on a sub-minute scale.

Electrical behavior was characterized by the discharge interruptions, IPI pattern entropy, response IPI distribution histograms and their properties. Sliding window histograms and entropy were calculated according to their classical definitions and for the same sequence of 40-s windows used in the entropy computation for direct comparison.

To study the influence of different stimuli IPI distributions on the preferred latencies, we built post stimulus time histograms (PSTHs), by plotting the experimental probability that a fish EOD occurs immediately after a stimulus EOD, set as time reference (Fig. 3B).

Analysis programs were developed in C++ language and Matlab (The Mathworks Inc., Natick, MA) and ran in free open source Ubuntu Linux 10.04 (Canonical Ltd., London, UK) based AMD64 3200****MHz PC-computers. Graphics were designed with Matlab (The MathWorks, Inc., Massachusetts, USA) and Gnuplot 4.2 (http://www.gnuplot.info).

## Results

### Non Stimulated Fish

To investigate how locomotor and electrical behavior changed over long periods we acquired continuous data for approximately 48 h from freely moving non-stimulated fish. All acquisitions were started immediately after introducing the fish in the measurement aquarium and were performed in complete darkness. Each experiment was started at a different time of the day. Fish were moved back to the maintenance aquarium with natural illumination at the end of the experiments.

**Figure 4:**
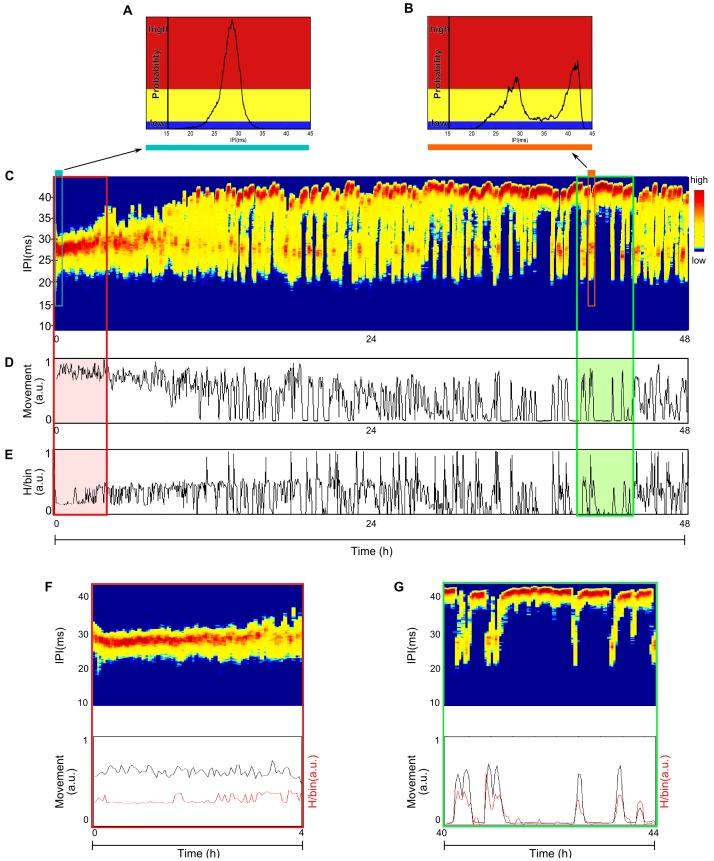
Non stimulated fish (48 h experiment). **A –** IPI histogram of the first 240(red), intermediate probable IPIs (yellow) and low probable IPIs (blue). In the beginning of the experiment the fish was more likely to fire 27 ms IPIs. **B –** IPI histogram for 240 s in the stationary period. The color code is the same as in A. During the stationary period the shape of the IPI distribution was bimodal with peaks in 28 ms and 40.5 ms. **C –** Sliding window histogram (SWH) of IPIs *versus* time. The histograms were calculated in 40 s windows and colors were assigned depending on the probability as explained in A. The sliding window histogram was built taking the colorcoded histogram for the first 40****s window and placing it in a vertical line in SWH, the second 40 s window colorcoded histogram was placed in the next vertical line in SWH and son on, allowing one to observe how IPIs evolve over time. There was a transient behavior, at the beginning of the recording, characterized by low average values of IPIs around 27.5 ms. After 10 h the behavior reached a stationary state that persists throughout the experiment: the IPIs distributions oscillated over time between the same two main peaks of IPIs, 27 ms and 42 ms. **D –** Inferred movement *versus* time. The fish movement was maximal during the initial transient and evolved to a stationary state that alternated with periods of vigorous activity and total absence of movement. **E –** Entropy *versus* time. During the transient period the entropy presented intermediate values. After the transient, the entropy oscillated between higher and almost zero values. **F –** Detail during the transient behavior. Above: Sliding window histogram of IPIs *versus* time. Below: Inferred movement (black) and entropy (red) *versus* time. At the beginning of the transient there are low IPIs values (high EOD frequencies) with constant values of entropy and restless movement. **G –** Same as F after habituating to the aquarium. In the stationary state, the IPIs oscillated between two mean values. Movement and entropy were entrained.

Two different behaviors were observed: a transient period characterized by low average IPI of approximately 27.5 ms (Fig. 4A) and a stationary one characterized by higher mean IPI (Fig. 4B). These persisted throughout the experiment, and the IPI distributions oscillated (Fig. 4C) between the same 2 main peaks, a sharper one corresponding to the higher IPI of 42 ms and another one corresponding to the lower IPI of approximately 27 ms (sharp peaks in Fig. 4A and B are represented in dark red, and IPIs with lower, but still significant, probability are represented in yellow in Fig. 4C).

The fish movement (Fig. 4D) was maximum during the initial transient and evolved to a stationary period that alternated between vigorous activity and absence of movement (LIA std near 0). The duration of the transient in the movement analyzes coincided with duration of the transient in EOD activity.

Analyzes of EOD patterns of entropy over time (Fig. 4E) revealed the same qualitative behaviors as described above. The locomotor activity peaks corresponded to high EOD variability timing, whereas the valleys corresponded to low variability timing, meaning that when the fish swam more vigorously its EO fired a greater variety of EOD patterns and vice-versa. When the entropy was near zero, the same pattern (periodic firing rate) was being fired.

During the transient period we found higher and generally constant values of locomotor activity and entropy (Fig. 4F). During the stationary period both locomotor activity and entropy entrained their peaks and valleys to express changes in the EOD rhythm (Fig. 4G). When the IPIs distribution over time was dominated by long IPIs (∼42 ms), the fish was stating still (locomotor activity ∼0) and firing periodic IPIs (entropy∼0).

All fish presented a much flatter distribution with a single mean when introduced in the new tank. This typical transient in response to the new environment lasted several hours, corresponding to a long relaxation time *T_R_*, defined as the time needed for the IPIs to reach the stationary state. For all fish recorded (N = 3) *T_R_* = (10±5) h. Despite quantitative differences all fish presented a final stationary state oscillating between 2 well defined mean IPIs. The duration of the EOD activity transient coincided with the duration of the transient in the locomotor activity analyzes and their peaks and valleys were entrained.

**Figure 5:**
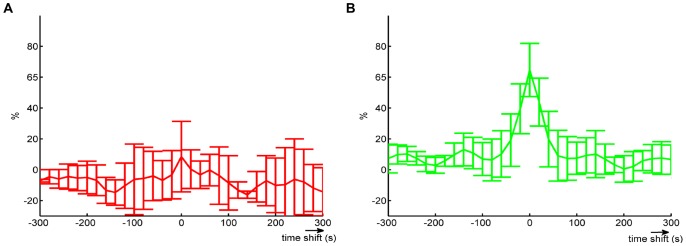
Cross-correlation between entropy and movement. **A –** Mean cross-correlation for the transient period. At lag 0, entropy and movement were only 10% correlated. The correlation remained low for all lags. Error bars indicate the standard deviation obtained from experiments with 3 fish. **B –** Cross-correlation for the stationary period. Entropy and movement were highly correlated (70%) at lag 0 and it quickly decreased.

The mean cross-correlation between EOD pattern entropy, and locomotor activity for the transient period (Fig. 5A) was much lower than that for the stationary period (Fig. 5B). Negative values of cross-correlation meant that the locomotor activity decreased when entropy increased, indicating that although the fish was swimming with less intensity, a great variety of EOD patterns were being fired. In the transient period, the cross-correlation remained approximately 10% for all lags, with a peak of 10% at lag 0. For the stationary period, there was a sharp peak of 70% at 0 lag that dropped abruptly to approximately 20% at the next lag and then remained at 10%.

There were a few spontaneous cessation of firing, defined as IPIs greater than twice the mean IPI, up to 5.3 s (Table 1). Spontaneous discharge interruptions were longer and more frequent in the stationary period, when fish were habituated to the new environment.

**Table 1:** Cessation of firing for 48 h non stimulated fish.

	Cessation of firing	
	Total number(% of total IPIs)	Duration (s)[min max]
Fish 1	34(0.0018)	[0.07 1.80]
Fish 2	114(0.0050)	[0.05 3.05]
Fish 3	186(0.0093)	[0.10 5.30]

### Stimulation Experiments

To compare the influence of distinct stimuli IPI distributions on the fish’s behavior, we used a real stimulus, pre-recorded from a fish without stimulation (black curve in Fig. 2). and a random (flat) distribution of IPIs between 15 and 20 ms (red curve in Fig. 2). Fish were subjected to 2 protocols consisting of 5 sessions of 30 min each. The sequence of the sessions in the first protocol was: first control recording (without stimulation; presented immediately after introducing a fish in the measurement aquarium), stimulation with a real IPI distribution, second control recording, stimulation with random IPI distribution, and the final third control recording. The second protocol was: first control recording (without stimulation; presented immediately after introducing a fish in the measurement aquarium), stimulation with random IPI distribution, second control recording, stimulation with a real IPI distribution, and the final third control recording.

**Figure 6:**
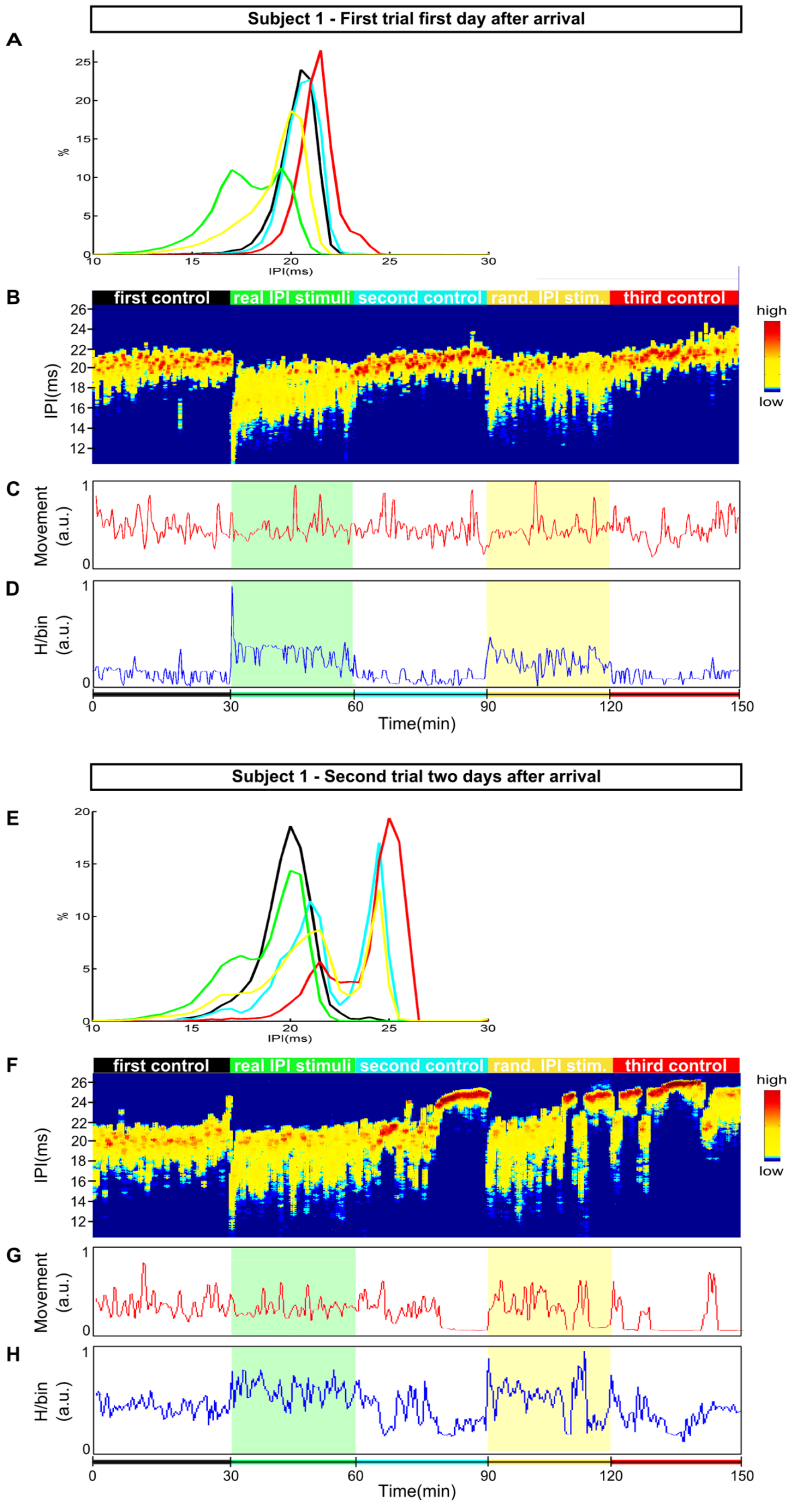
Experiments with stimulation. The protocol was a sequence of 5 sessions (30 min each) as follows: first control recording (without stimulation)(black), stimulation with a real distribution of IPIs (green), second control (blue), stimulation with random distribution of IPIs (yellow), and a final control (red). **A-** Histograms of the response IPIs for each session: first, second and third controls in black, blue and red respectively. Response to real stimuli in green and to random one in yellow. In the first 30 min, fish discharged mostly 20.5 ms IPIs. In response to real IPI stimuli, the IPI distribution was bimodal with 2 equally probable values: 17 ms and 19.5 ms. In the second control session the IPI distribution changed presenting a single sharp peak in 21 ms. The distribution in response to a random stimuli was broader than for the control session with a single peak in 20 ms. The fish reacted to the third control session firing mostly longer IPIs with a peak in 21.5 ms. **B –** Sliding window histogram of IPIs *versus* time. High (low) probabilities are shown in red (blue). The same colorcode (red/yellow/blue) associated with high/intermediate/low probability explained in Fig. 5 is used here. **C –** Inferred movement, and **D –** entropy *versus* time. Fish reacted to both stimuli by decreasing the IPIs values (increasing the EODs frequency) and increasing its variability from 19–22 ms to 14.5–21 ms. During random stimuli (yellow bar) fish presented a slight relaxation with the peak of IPIs tending to higher values over time from 20 ms to 21.5 ms as well as in the third control session from 21 ms to 23 ms. The fish was restlessly moving throughout the experiment with no simple relation to the stimulation sessions. The entropy clearly increased (decreased) when both stimuli were turned on (off). **E –** Same as A but 2 weeks later. For the first 30****min, the IPI distribution presented a single sharp peak in 20 ms (black). For the rest of the sessions the IPI distribution were bimodal differing in the peak values. The fish fired shorter IPIs (17.5 ms) in response to a real IPI stimuli session (green). The IPI distributions in response to the second control (blue) and random stimuli (yellow) were very similar with peaks around 21 ms and 24.5 ms. For the last control session fish fired longer IPIs compared to the previous sessions with peaks in 21.5 ms and 25 ms. **F-H** **D-F –** same as **A-C B-D** but for an experiment performed 2 weeks later with the same individual. The same qualitative behavior was found with the remarkable exception that during the stimulation with a random distribution fish presented several epochs characterized by: very high IPI values around 24.5 ms, absence of movement, and very low entropy values, probable sleep-like state that was expected only during control sessions.

For subject A, subjected to the first protocol, the mean IPI distribution changed from a sharp peak at 20.5 ms (black curve in Fig. 6A) for the first control session to broader bimodal IPIs distribution with peaks at 17 ms and 19.5 ms for the stimulus session with real IPIs (green curve in Fig. 6A). In the second control session (blue curve), the IPIs distribution had a shape similar to that for the first control session (black curve). The response IPI distribution for the random stimuli session presented a single sharp peak at 20 ms (yellow curve), in contrast to a bimodal distribution observed in the real IPI stimuli session (green curve). The probability of discharging shorter IPIs (17 ms and 19.5 ms) was higher for the first stimuli session (real IPI stimuli) compared to the second stimuli session (random) (20 ms). In the third control session, the fish discharged longer IPIs (lower frequency) with a sharp peak at 21.5 ms (red curve).

As soon as the stimulus was turned on, the fish reacted by shortening its IPIs and increasing the variability, abruptly changing the range of the IPIs from 19–22 ms to 11–20.5 ms (beginning of the green bar, yellow and red IPIs in Fig. 6B). A broad range (14.5–21 ms) of highly probable IPIs persisted throughout the stimulation session. Similar behavior was observed for the random stimuli session, but the range of IPIs was slightly narrower than that for the real stimuli session (14–21 ms; beginning of the yellow bar, yellow and red IPIs in Fig. 6B).

Immediately after the stimulus was turned off in both real and random stimuli sessions, the mean IPIs became longer (20 ms and 21 ms), and the variability decreased to 18.5–21 ms and 18.5–21.5 ms, respectively. Throughout the second and third control sessions, the IPIs gradually became longer (∼23 ms), particularly in the second half of the third control session.

The entropy and locomotor activity (Fig. 6C and D) did not show the same relationship observed during the stationary period in unstimulated fish (Fig. 4G). The entropy did not always increase when the fish swam more vigorously. For example, approximately 10 min after the beginning of the second stimuli session (yellow shade in Fig. 6C and D), there was a high value of movement but low entropy. In this case, the fish increased its movements while the EO was firing the same IPI patterns.

In experiments performed two weeks after the fish arrived at the laboratory, it fired longer IPIs. The histogram for the first control session showed a peak at 20 ms (black curve in Fig. 6E) and a range of 16–22 ms (first 28 min in Fig. 6F), but in the final minutes of this session the IPIs became longer (19–24.5 ms). During the first stimulation session (real IPI distribution), the probability of firing shorter IPIs increased, and a new peak was observed at 17.5 ms (green curve in Fig. 6E). In the second control session and the random stimuli session, the EO fired with predominantly 2 IPIs: 21 ms and 24.5 ms (blue and yellow curves in Fig. 6E). The EO fired at longer IPIs in the last control session compared to the previous sessions, specifically IPIs of 21.5 ms and 25 ms (red curve in Fig. 6E).

Fish typically reacted differently when subjected to the stimulation protocol for the first time, a few days after arriving at the laboratory (Fig. 6A–D), and after ∼2 weeks (Fig. 6E–H). In the second trial, the same increase in the EOD frequency, entropy, and movement was observed during all stimuli sessions (green and yellow bars in Fig. 6A, B, E, and F) as that observed during the first trial (conducted at the time of introduction). However, a distinct reaction was observed in the second half of the random stimuli session in the second trial (yellow bar in Fig. 6B and F); the EOD frequency and variability during this part of the session were lesser than that during the beginning of this stimulation session and the second stimulus session in the first trial. Similar tendency was observed for the locomotor behavior (Fig. 6G), as well as for entropy (Fig. 6H).

Interestingly, 2 weeks after arriving at the lab, both entropy and movement vanished, meaning that the fish was standing still and periodically firing long IPIs for periods up to 10 min when the EOD frequency decreased at the end of the random stimuli session (yellow bar in Fig. 6 G and H). This behavior is similar to that found in the stationary period in a non-stimulated fish, with the entropy and movement highly correlated (65%).

The fish J subjected to protocol 2 (Fig. S1) presented similar behaviors than those described above using protocol 1.

**Figure 7:**
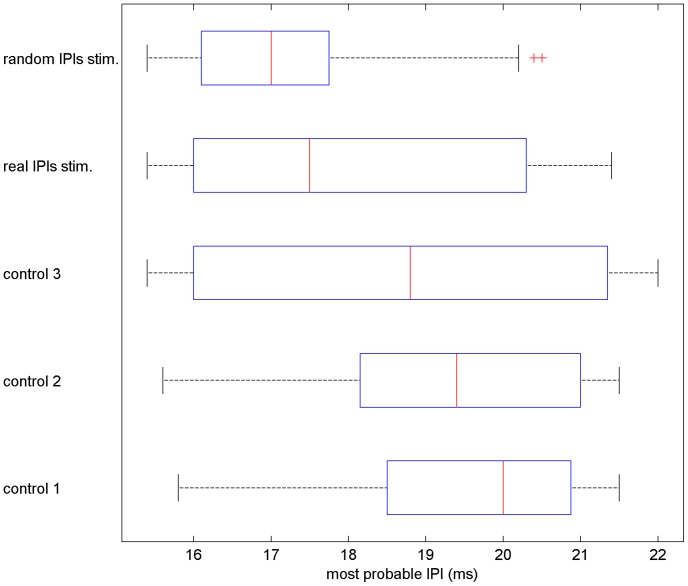
Most probable IPIs for control and stimuli sessions. The most probable IPIs discharged during the stimuli sessions were smaller than those for the control sessions, the medians were 17.0(Wilcoxon p<0.05). However, there were no differences among the most probable IPIs discharged during the control sessions (no stimuli sessions; Friedman p>0.05).

For all fish in both protocols, the most probable IPIs in response to the stimuli sessions were shorter than those to the random stimuli ones (Fig. 7). Fish discharged shorter IPIs (17.0 ms median) during the real stimuli sessions compared to those for the random stimuli sessions (17.5 ms median) (Wilcoxon p<0.05). There were no differences in the response to the control sessions (Friedman p>0.05). The medians for the control sessions 1, 2 and 3 were, respectively, 18.4 ms, 18.8 ms and 19.4 ms. The most probable IPIs in response to real stimuli session were equal to or shorter than those to the random stimuli session (Fig. 7).The highest difference was (24.4%), discharging more often 17.2 ms IPIs for the real stimuli session and 21.4 ms IPIs for the random stimuli session, and the lowest (1.2%) was 16.4 ms and 16.6 ms IPIs for real and random stimuli session respectively.

**Figure 8:**
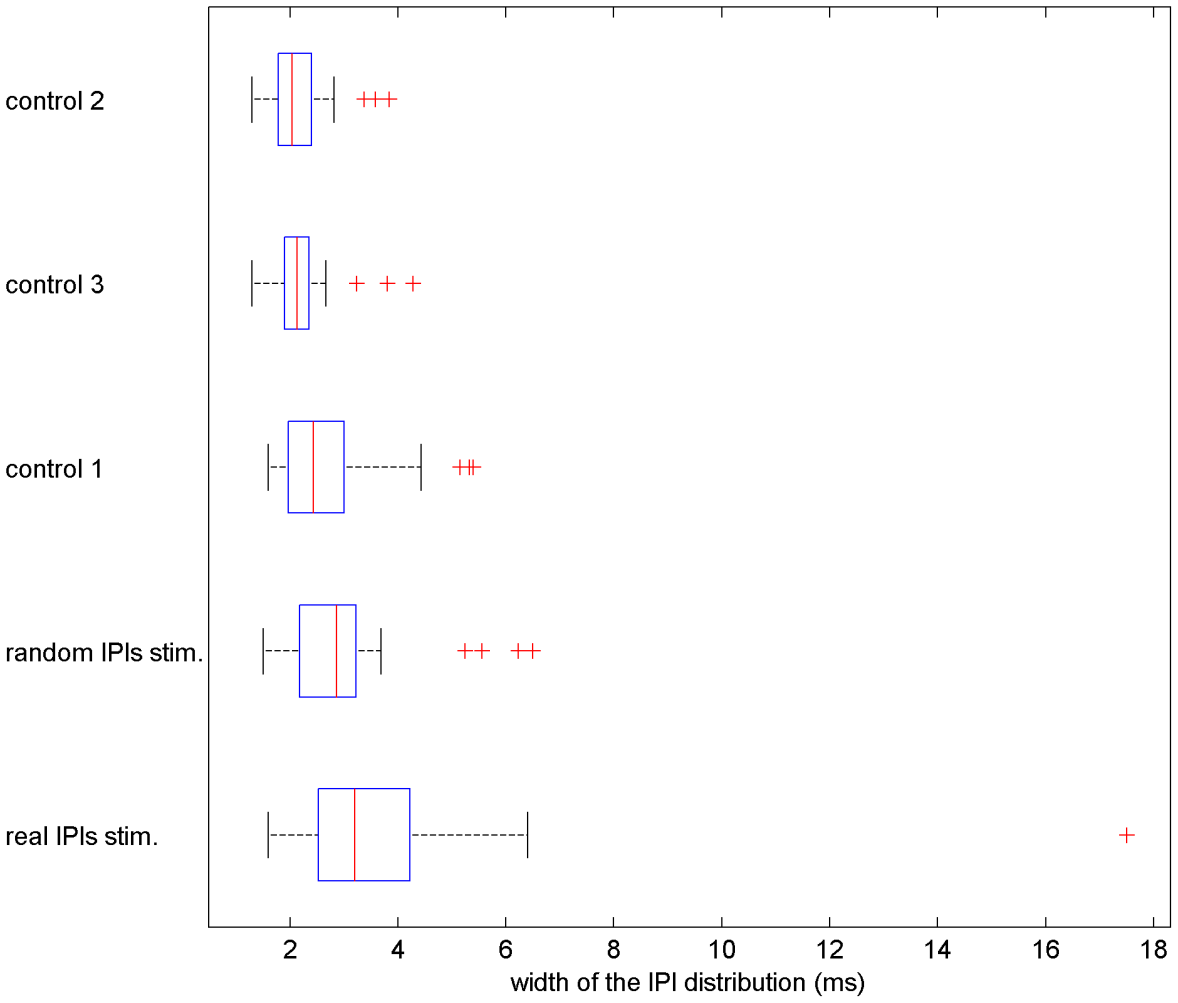
Average width of IPI distributions. Fish discharged greater variety of IPIs (wider distributions) during the stimuli sessions compared to the control sessions, medians were 3.2 ms and 2.86 ms for the real and random IPI sessions and 2.43 ms, 2.13 ms and 2.04 ms for control session 1, 3 and 2 respectively. Between the stimuli sessions, the average width of the IPI distributions for the real IPI sessions were wider (Wilcoxon p<0.05). For the control sessions (Friedman p<0.05), post-hoc analyzes revealed that control 1 was statistically different than control sessions 2 and 3. The width of the distribution were calculated as the 2–98 percentile measure in 40****s sliding windows.

Fish vary their firing rate as well as the IPI variability over time. To quantify these changes in addition to the most probable IPIs we computed the average of spread (2–98 percentile) of the IPI distribution for all sessions (individually) by calculating the width of the IPI distribution for each 40****s window and taking the average. Fish presented wider IPIs distributions when stimulated compared to those when non-stimulated (Fig. 8). Fish responded differently to the real control sessions than to the random stimuli ones, discharging greater variety of IPIs (3.2 ms median) in response to the real control sessions compared to the random stimuli ones (2.86 ms median; Wilcoxon p>0.05).The highest differences between the spread of the real and random stimuli sessions were 169.2% and 48.4%. Among the control sessions (Friedman p<0.05), post-hoc analyzes showed that control 1 (2.43 ms median) was statistically different than control sessions 2 (2.04 ms median) and 3 (2.13 ms median). Control 1 presented higher width of IPI distribution in 17 out of 23 experiments.

**Figure 9:**
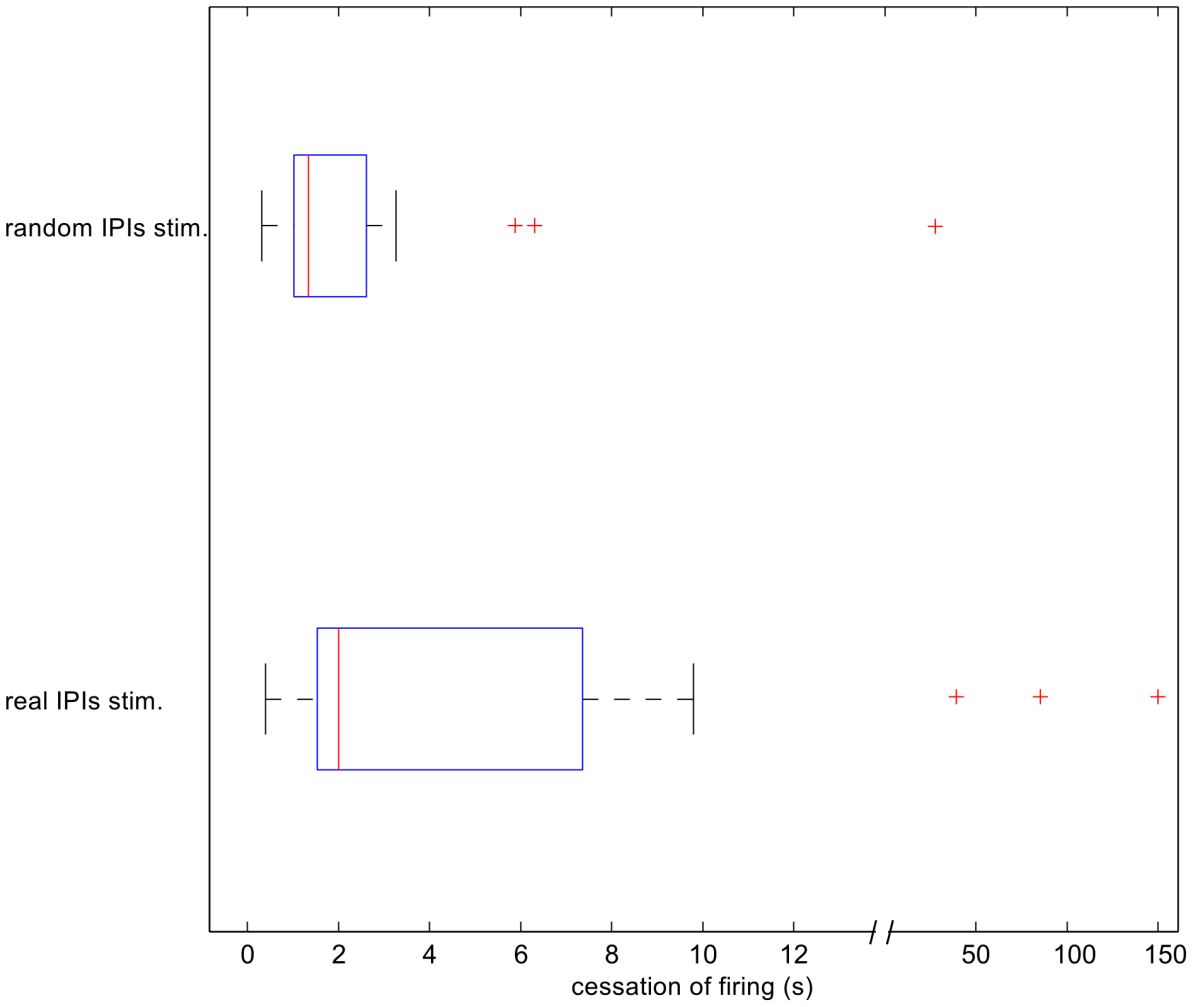
Cessation of fire. Fish presented longer cessation of fire during the real stimuli sessions compared to those for the random stimuli ones (Wilcoxon p<0.05). The longest cessations of fire lasted 150 s for the real stimuli sessions and 43 s for the random stimuli ones. The medians were 2.0 s and 1.3 s for the real stimuli sessions and random stimuli sessions, respectively. Cessation of firing was defined those IPIs 2 times longer than the mean IPI.

Fish usually presented spontaneous cessation of firing (defined as IPI >2×mean IPI). Fish showed longer cessation of firing when subjected to real stimuli compared to that for the random stimuli (Fig. 9). A single fish (the smallest one, ∼15 cm) stopped firing for over 2 min when the real IPI distribution stimulus was turned on, whereas, the longest cessation of firing in response to a random stimulus was 43 s. Spontaneous brief interruptions were less likely to occur during control sessions (Table 2) compared to those for stimuli sessions.

**Table 2:** Cessation of firing.

Mean%±StD cessation of firing during:				
First control sessions	Real IPI stimulisessions	Second controlsessions	Random IPI stimulisessions	Third controlsessions
0.01±0.03	*0.18±0.47*	*0.01±0.02*	*0.12±0.27*	0.01±0.02

**Figure 10:**
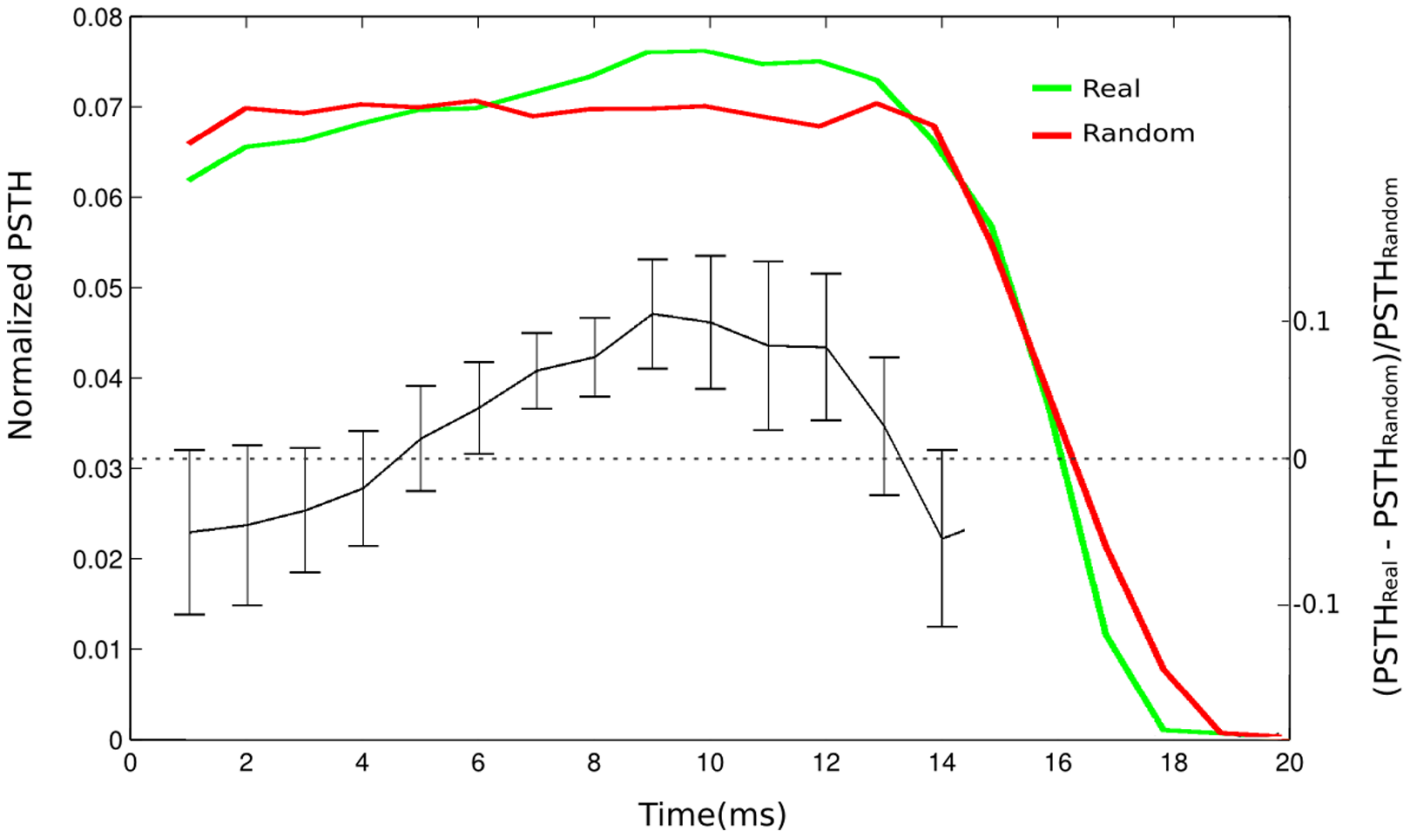
Normalized Post Stimulus Time Histograms (PSTH). The PSTH for stimuli from a random distribution of IPIs (red) presents a flat shape up to the limit of the mean IPI (∼17 ms), whereas the PSTH for a real distribution of stimuli IPIs presents a peak probability of approximately 10 ms. The PSTH change (black) when the random stimuli distribution is replaced by real stimuli shows an average decrease of probability (∼5%) in the 0–5 ms-range and the probability in the range of 6–12 ms increases ∼10%. Error bars indicate the standard deviation, obtained from experiments with 8 fish. The changes correspond to 3% to 5% of the total number of EODs and is evidence that fish discriminate between real and random stimuli distributions (ANOVA, p<0.002).

To address whether fish manifested different latencies when subjected to real or random IPI distributions we built normalized Post Stimulus Time Histograms (PSTH), where we plotted the experimental probability that a fish EOD occurs immediately after an artificial EOD (set as time reference) (Fig. 3 B) for both real and random IPI stimuli distributions (Fig. 10). That is, after receiving a stimulus pulse, we questioned how long will it take for the fish’s EO to discharge.

After an artificial EOD from a random stimuli distribution the fish presented a flat EOD probability up to the limit of the average stimulus IPI (∼14 ms, Fig. 10). However, after an artificial EOD from a real stimuli distribution, the fish EOD probability was lower than that for the random stimulus in the 1- to 5-ms interval and higher in the 6- to 12-ms interval. This indicates that fish were more likely to wait longer to fire after receiving a pulse stimulus from a real IPI distribution than after a random one. PSTHs for experimental sessions with the real stimuli distribution revealed a preference for firing 3% to 5% of the total EOD number later than that observed in PSTHs for experimental sessions using the random stimuli distribution.

In order to generalize the analyses we calculated PSTHs from both conditions for 8 fish, integrated them in steps of 1 ms, and computed how much the PSTH for sessions with the real stimuli distribution deviated from the flat PSTH for sessions with the random stimuli distribution, i.e., we calculated (PSTHReal - PSTHRandom)/PSTHRandom for each 1 ms bin from 1 to 14 ms (Fig. 10). From the average behavior of 8 fish, it was clear that in response to a real stimuli distribution, EOD probability in the first 1–5 ms was lower and in 6–12 ms was higher than that in response to random stimuli (ANOVA, p<0.002).

## Discussion

Pulse-type weakly electric fish allow non-invasive access to central nervous system patterns involved in 2 interesting but incompletely understood tasks: electrolocation and electrocommunication [1], [2], [52]. However, due to experimental difficulties in dealing with a complex spatio-temporal electrical pattern generated by a freely moving fish, most studies on the interaction between fish with dimorphic pulse shapes and different pulse amplitudes used non-realistic waveforms as stimuli [18], [20], [53].

We developed an apparatus that enabled us to reliably detect and record in real time the EOD timestamps of freely swimming fish in 3 dimensions over several days using a cubic arrangement of electrodes and dedicated real-time software. Real-time software allowed us to stimulate the fish in a controlled way, mimicking the pulses of a conspecific but according to a distribution of IPIs chosen by the experimenter. Our setup accepts any kind of waveforms and stimuli IPI distributions, allowing one to perform experiments with most species of electric fish [42]–[44] and to study how a given specie would behave in the presence of different species.

Our 48****h experiments with non-stimulated fish revealed that introducing a fish into a novel environment (the acquisition aquarium) triggered a transient exploratory behavior characterized by short IPIs (high frequency EODs) as well as intense movement around the aquarium. After a long period (the order of 10 h) a final stationary period with the EODs oscillating between 2 main frequencies was reached.

It was known that electrical behavior and movement can be associated [53] at least for short periods. Acoustic noise or intense light startled the fish and the EODs momentarily increased and the fish swan away. Nevertheless it was not expected that movement and electrical behavior (entropy of EODs patterns) could be related for such long periods. Surprisingly, after the transient period, movement and entropy were entrained for hours, with 70% correlated. That is, most EODs contain information about electrolocation.

During stimulation experiments turning on/off the stimulus aroused almost all fish in opposition to the data described by Bullock [53]. A plausible explanation is the difference in the waveform used in these experiments. We used the waveform of *G.* *carapo*, whereas Bullock used rectangular pulses.

In experiments with stimulation, entropy and movement were not strongly correlated, except for a few periods of possible behavioral sleep. That is, changes in the EODs patterns are due not only to fish movement but also due to the encoding of distinct sources of information. In these controlled experiments, we know that fish are freely swimming and receiving stimuli. Thus, we hypothesize that the additional entropy could correspond to electrocommunication.

There were also low correlation values of entropy and movement for the control sessions after turning off the stimulus. This is likely because the fish were using their EODs not only for electrolocation (which would provide high correlation), but also must be trying to find the suddenly disappeared conspecific that was communicating with them.

Both stimuli with real and random IPI distribution triggered increases in the mean EOD frequency, in the IPI distribution width and in the cessation of firing. However, fish did not respond equally to both stimuli. Fish were more likely to discharge shorter IPIs (hig frequency EODs), wider range of IPIs and longer cessation of fire in response to real IPI stimulation sessions than to random IPI stimuli sessions for both protocols. Fish behavior was similar when non-stimulated (control sessions) with only one exception, the variability of IPIs during the first control sessions were bigger than those for the second and third ones due to the novelty of the environment in control 1.

Real and random stimuli have the same waveform and amplitude, differing only in terms of IPI distributions (Fig. S2). In the flat random distribution all IPIs occur with the same probability and there are no causalities. Conversely, in the real distribution, some IPI values are more likely to occur than others. Additionally, the sequence in which the real IPIs appeared are truly from a real fish, and not randomly assigned by computer. We hypothesized that the order that each IPI is fired, that is, the causality, play an important role in communication and fish are able to identify unrealistic sequences of IPI patterns. In future work, we will investigate how these sequences vary in response to different stimuli and behavioral contexts. Specifically, we will address how different the order in which the IPI patterns are if the fish are isolated, socially naïve, habituated, or not in a new environment.

To analyze the differences in the EOD timming under stimulation, we compared several fish EOD occurrence probabilities immediately after being exposed to a real or a random stimulus in PSTHs. The probability of an EOD after a pulse coming from a random distribution of IPIs is constant over time. However, the probability of an EOD after a stimulus pulse coming from a real distribution of IPIs is slightly reduced (3–5% of the pulses) for short times and increased for longer times. This result is another evidence that fish are able to discriminate between these 2 distributions. Moreover, the result obtained with the real stimuli in a freely moving fish agrees with those preliminary found in an experiment with 2 real fish hidden in plastic pipes [21].

It has been hypothesized that electric fish might separate their own electric pulses from external ones [54], [55]. It was recently shown, in brain slices, that spherical neurons at the fast electrosensory path of *Gymnotus omarorum* have an intrinsic refractory time of ∼10 ms which is about half the average IPIs in those fish [56]. In the same work there are also evidences, from chronically implanted fish experiments, indicating that the animal uses this refractory time to minimize the interference of conspecific pulses in the sensory pathway.

Our PSTH results for freely moving fish revealed a preference for discharging after half of the average IPI of the conspecific (stimuli from real distribution), which indicates after the conspecific refractory period. Thus, our results suggest that in our experimental conditions the fish changes the timing of the EOD to be detected by the conspecific and would have to delay its next EOD to avoid the refractory period of its own spherical neurons.

We propose that a fish estimates the refractory period of a conspecific and chooses whether to discharge within this period. For example, to electrolocate without being detected it would be interesting to discharge within the refractory period of the conspecific; whereas to communicate a fish would discharge out of the conspecific refractory period. If this is the case, one must take in account both refractory periods when analyzing communication between fish.

To confirm this hypothesis and to estimate the refractory period in the freely moving fish *Gymnotus carapo*, we are currently planning real-time experiments in which stimulus pulses will be delivered according to the fish’s own EODs, as well as experiments with 2 fish in the same tank and software techniques to classify and split their pulses.

## Supporting Information

Figure S1 **Experiments with stimulation using the second protocol. A** – Sliding window histogram of IPIs *versus* time. The histogram was also calculated in 40 s windows and colors are assigned depending on the probability as explained in Fig. 3A. The second protocol was: first control session (without stimulation; black bar), stimulation using the random IPI distribution (yellow bar), second control (blue bar), stimulation using the real IPI distribution (green bar) and third control (red bar). When the random stimulus was turned on (beginning of the yellow bar), the fish reacted in the first 10 min, shortening its IPIs firing in the range of 15.5–20 ms. In the last 20 min, the IPIs became longer ∼20 ms changing the rang of IPIs to 17–20.5 ms. Nevertheless, when stimulated with the real IPI distribution, a broad range (16–21 ms) of highly probable IPIs persisted throughout the stimulation session. Responses to real stimuli sessions usually presented broader IPIs distributions than those to random stimuli in both protocol (for protocol 1 see Fig. 8 B and F). For all control session the mean IPI was longer ∼21.5 ms compared to the stimulation sessions. **B** – Inferred movement, and **C** – entropy versus time. The fish was restlessly moving throughout the experiment, specially during stimulation sessions. The entropy showed a small increase for the stimulation sessions. Increased (decreased) in entropy and movement were not entrained.
(TIF)

Figure S2 **Real and random stimuli over time – Sliding window histogram of IPIs** ***versus*** **time.** The histograms were also calculated in 40 s windows and colors are assigned depending on the probability as explained in Fig. 3A. In the real fish IPI distribution the most probable IPIs (in red and yellow) change over time mostly from 15 ms to 20 ms and in the final minutes from 20 ms to 21.5 ms. In the random IPI distribution, all IPIs between 15 ms and 20 ms occurred with the same probability avoiding possible causalities.
(TIF)
