# Supplementary figures and images for: Automatic Realistic Real Time Stimulation/Recording in Weakly Electric Fish: Long Time Behavior Characterization in Freely Swimming Fish and Stimuli Discrimination
Source: PLoS One. 2014 Jan 6;9(1):e84885. doi: 10.1371/journal.pone.0084885 (PMC3882270; doi:10.1371/journal.pone.0084885)

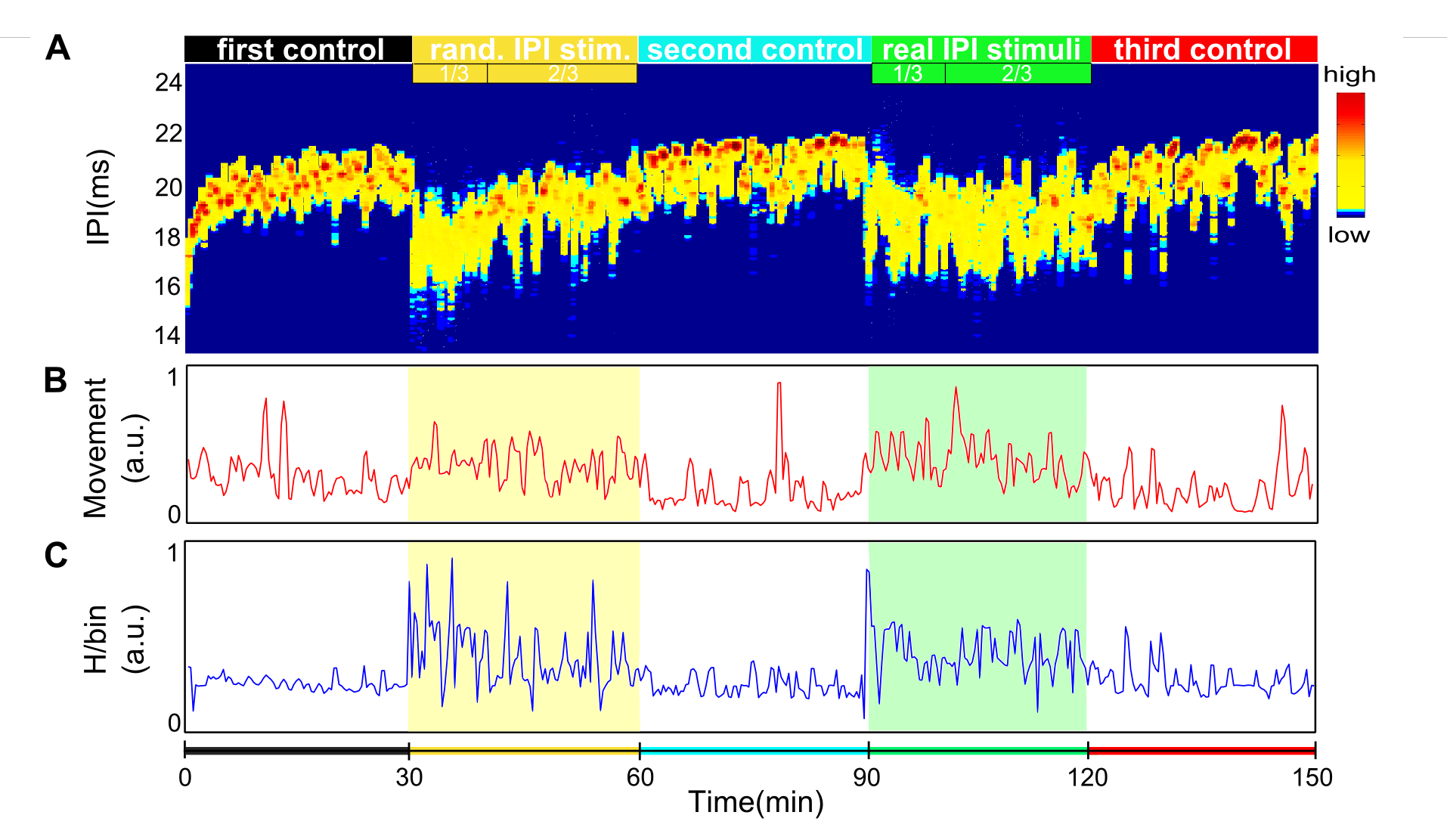

Supplement: Figure S1 — Experiments with stimulation using the second protocol. A – Sliding window histogram of IPIs versus time. The histogram was also calculated in 40 s windows and colors are assigned depending on the probability as explained in Fig. 3A. The second protocol was: first control session (without stimulation; black bar), stimulation using the random IPI distribution (yellow bar), second control (blue bar), stimulation using the real IPI distribution (green bar) and third control (red bar). When the random stimulus was turned on (beginning of the yellow bar), the fish reacted in the first 10 min, shortening its IPIs firing in the range of 15.5–20 ms. In the last 20 min, the IPIs became longer ∼20 ms changing the rang of IPIs to 17–20.5 ms. Nevertheless, when stimulated with the real IPI distribution, a broad range (16–21 ms) of highly probable IPIs persisted throughout the stimulation session. Responses to real stimuli sessions usually presented broader IPIs distributions than those to random stimuli in both protocol (for protocol 1 see Fig. 8 B and F). For all control session the mean IPI was longer ∼21.5 ms compared to the stimulation sessions. B – Inferred movement, and C – entropy versus time. The fish was restlessly moving throughout the experiment, specially during stimulation sessions. The entropy showed a small increase for the stimulation sessions. Increased (decreased) in entropy and movement were not entrained. (TIF) [file pone.0084885.s001.tif]

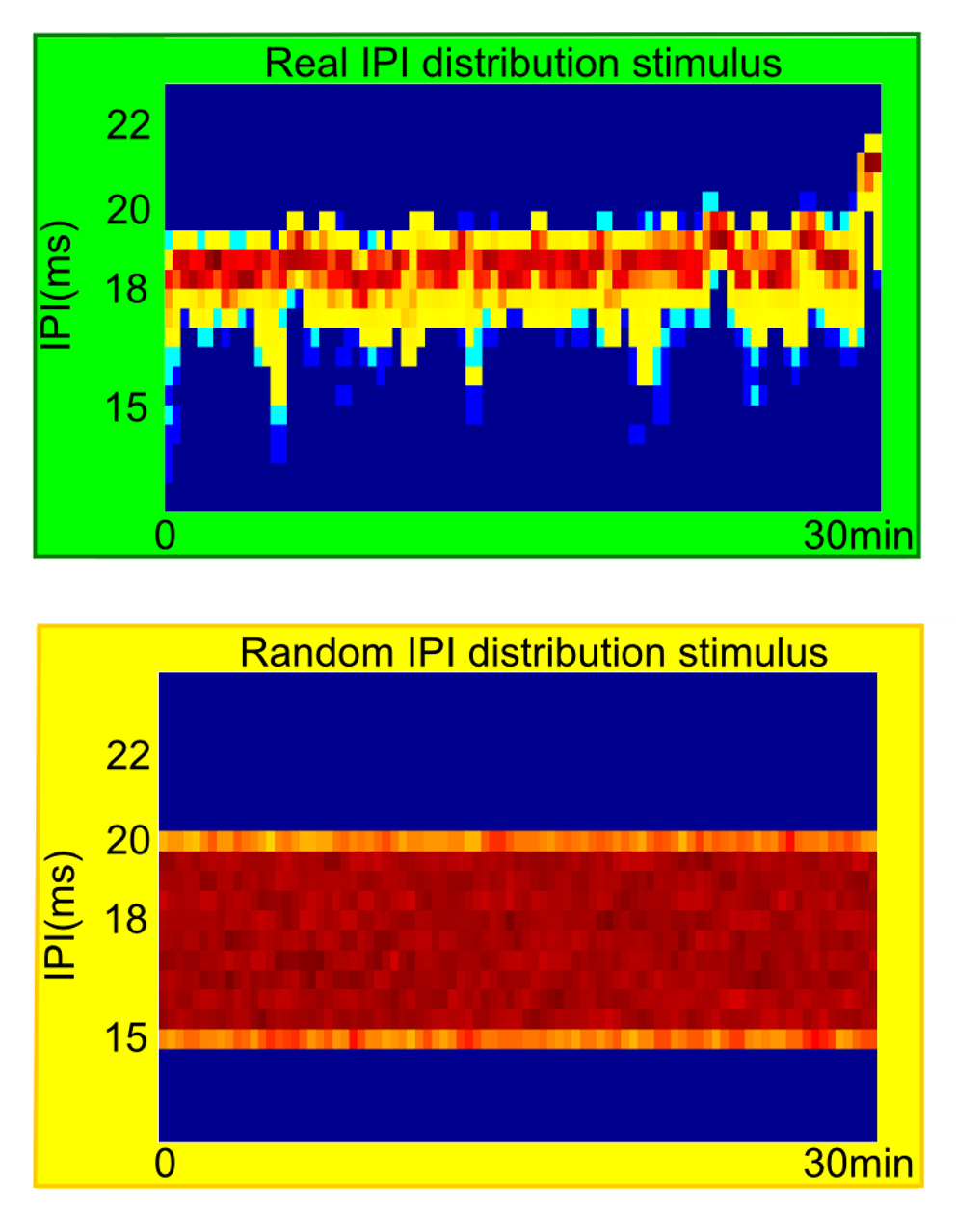

Supplement: Figure S2 — Real and random stimuli over time – Sliding window histogram of IPIs versus time. The histograms were also calculated in 40 s windows and colors are assigned depending on the probability as explained in Fig. 3A. In the real fish IPI distribution the most probable IPIs (in red and yellow) change over time mostly from 15 ms to 20 ms and in the final minutes from 20 ms to 21.5 ms. In the random IPI distribution, all IPIs between 15 ms and 20 ms occurred with the same probability avoiding possible causalities. (TIF) [file pone.0084885.s002.tif]
